# Bis[4-(diphenyl­methyl­eneamino)phen­yl]methanone

**DOI:** 10.1107/S1600536810016375

**Published:** 2010-05-12

**Authors:** Sylvain Bernès, Guadalupe Hernández, Roberto Portillo, Sandra Cruz, René Gutiérrez

**Affiliations:** a DEP Facultad de Ciencias Químicas, UANL, Guerrero y Progreso S/N, Col. Treviño, 64570 Monterrey, NL, Mexico; b Laboratorio de Síntesis de Complejos, Facultad de Ciencias Químicas, Universidad Autónoma de Puebla, AP 1067, 72001 Puebla, Pue., Mexico; c Departamento de Ingeniería Química, Universidad Politécnica de Tlaxcala, Calle 21, no. 611, Col. La Loma Xicohténcatl, Tlaxcala, Tlax., Mexico

## Abstract

The title mol­ecule, C_39_H_28_N_2_O, is a well known dendron used in the synthesis of phenyl­azomethine dendrimers. The central benzophenone core is twisted, as expected, due to hindrance between H atoms: the dihedral angle between core benzene rings is 54.49 (5)°, identical to that of the stable polymorph of benzophenone (56°). For the same reason, phenyl groups substituting imine C atoms make a large dihedral angle, although similar for each imine: 71.83 (6) and 67.64 (5)°. The six aromatic rings in the mol­ecule thus seem to be quite randomly oriented, and such an arrangement is not favorable for efficient stacking inter­actions in the crystal. The same behaviour is observed in the vast majority of diphenyl­imino-containing organics. The low triclinic crystal symmetry may be a consequence of these features.

## Related literature

For the use of the title mol­ecule in the synthesis of dendritic systems, see: Higuchi *et al.* (2001 ▶); Takanashi *et al.* (2004 ▶); Yamamoto & Higuchi (2004 ▶). For the structure of benzophenone, see: Fleischer *et al.* (1968 ▶); Kutzke *et al.* (2000 ▶). For related structures including the diphenyl­imino fragment, see: Appel *et al.* (1985 ▶); Buhmann *et al.* (1993 ▶). For geometrical analysis using the Cambridge Structural Database, see: Bruno *et al.* (2002 ▶).
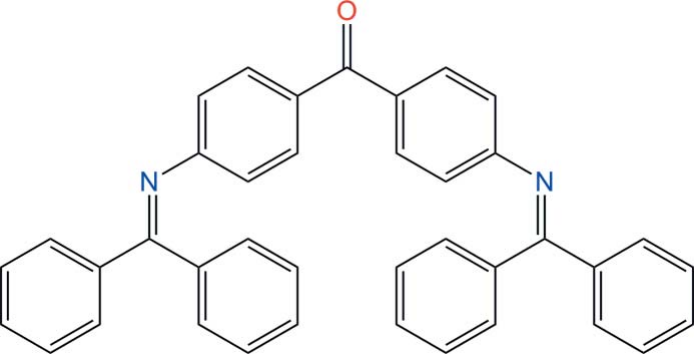


## Experimental

### 

#### Crystal data

C_39_H_28_N_2_O
*M* *_r_* = 540.63
Triclinic, 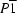

*a* = 11.1723 (10) Å
*b* = 11.3487 (13) Å
*c* = 13.2331 (15) Å
α = 103.121 (9)°
β = 105.170 (8)°
γ = 108.746 (8)°
*V* = 1441.9 (3) Å^3^
*Z* = 2
Mo *K*α radiation
μ = 0.08 mm^−1^
*T* = 296 K
0.55 × 0.28 × 0.24 mm

#### Data collection

Bruker P4 diffractometer
6892 measured reflections
5865 independent reflections
4471 reflections with *I* > 2σ(*I*)
*R*_int_ = 0.018
3 standard reflections every 97 reflections  intensity decay: 1%

#### Refinement

*R*[*F* ^2^ > 2σ(*F* ^2^)] = 0.042
*wR*(*F* ^2^) = 0.113
*S* = 1.03
5865 reflections
380 parameters
H-atom parameters constrained
Δρ_max_ = 0.19 e Å^−3^
Δρ_min_ = −0.14 e Å^−3^

### 

Data collection: *XSCANS* (Siemens, 1996 ▶); cell refinement: *XSCANS*; data reduction: *XSCANS*; program(s) used to solve structure: *SHELXS97* (Sheldrick, 2008 ▶); program(s) used to refine structure: *SHELXL97* (Sheldrick, 2008 ▶); molecular graphics: *Mercury* (Macrae *et al.*, 2008 ▶); software used to prepare material for publication: *SHELXL97* .

## Supplementary Material

Crystal structure: contains datablocks I, global. DOI: 10.1107/S1600536810016375/fl2303sup1.cif

Structure factors: contains datablocks I. DOI: 10.1107/S1600536810016375/fl2303Isup2.hkl

Additional supplementary materials:  crystallographic information; 3D view; checkCIF report

## Figures and Tables

**Fig. 1.**
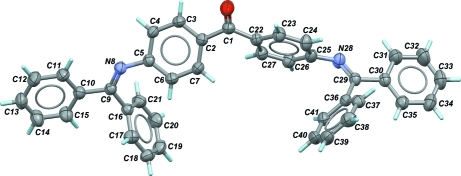
Molecular structure of the title compound, with 50% probability level displacement ellipsoids for non-H atoms.

## supplementary crystallographic information

### Comment

The title benzophenone derivative has been widely employed as a dendron in the synthesis of phenylazomethine dendrimers (DPAs), mostly in the group of Yamamoto at the Keio University (Higuchi *et al.*, 2001; Takanashi *et al.*, 2004; Yamamoto & Higuchi, 2004). This group and others reported on the preparation of a vast array of supramolecular entities with interesting properties. We became interested in preparing this dendron by using microwave heating, given that it is becoming an important method in laboratories worldwide: it is an environment-friendly technique for the efficient syntheses of organic molecules. The main advantages of microwave-assisted organic synthesis are shorter reaction times, minimum waste and generally higher yields, operational simplicity as well as reduction of thermal degradative byproducts along with cleaner work-up. As expected, better yields were obtained and we realized that, surprisingly, the crystal structure had not been reported so far.

The molecule (Fig. 1) crystallizes in the low symmetry space group *P*1. The imine bond lengths, 1.2813 (18) and 1.2784 (19) Å, are as expected, however, N atoms significantly deviate from trigonality. Large C═N—C angles are observed, 127.61 (12) and 123.09 (13)°, probably because of the steric repulsion between the central benzophenone benzene rings and the diphenylmethylene groups. The central benzophenone displays a twisted conformation, the dihedral angle between benzene rings being 54.49 (5)°. This value is indeed close to that reported for benzophenone, 56° (orthorhombic phase, Fleischer *et al.*, 1968) or 64° (metastable monoclinic phase, Kutzke *et al.*, 2000). This conformation avoids any intramolecular H···H contacts. In the same way, diphenyl groups bonded to imine C atoms are twisted, by 71.83 (6)° (diphenyl group at C9) and 67.64 (5)° (diphenyl group at C29). These angles are common for diphenylimino-containing organics (range of angles retrieved from the CSD : 57 to 90°; CSD, version 5.31 with all updates; Bruno *et al.*, 2002).

As a whole, the six rings in the molecule seem to be randomly oriented. This chaotic arrangement is consistent with the low crystal symmetry, and does not favor π···π or C—H···π interactions in the crystal structure. For example, the shortest intermolecular separation between centroids of two rings is 4.45 Å. The calculated packing index is indeed low for this polyphenyl molecule, 0.672. A search in the CSD for organic molecules containing the Ph_2_C═N fragment shows that more densely packed crystals in this class of compounds are scarce. For 151 hits, only two structures present symmetry-related diphenylimino groups with phenyl rings separated by less than 3.9 Å (Appel *et al.*, 1985; Buhmann *et al.*, 1993).

### Experimental

A modified procedure for improved synthesis of the title compound was used. The Higuchi's route (Higuchi *et al.*, 2001; see compound 'dendron G2' in this paper) consists of the condensation between benzophenone and 4,4'-diaminobenzophenone in presence of DABCO (1,4-diazabicyclo[2.2.2]octane) and TiCl_4_, in chlorobenzene. In the original synthesis, the mixture was heated at 398 K for 24 h to afford dendron G2 in 48% yield. In place of thermal activation, we performed a microwave-assisted synthesis in a monomode MIC-1 oven (Tekno-lab, S.A.) with maximum power output of 600 W. Irradiation was applied for 20 min., affording the title compound with an enhanced yield of 65% after silica gel column chromatography (ethyl acetate:hexane = 1:5). Single crystals were obtained by slow evaporation of the eluate at 298 K.

### Refinement

All H atoms were placed in idealized positions and refined as riding to their carrier C atoms, with bond lengths fixed to 0.93 Å. Isotropic displacement parameters were calculated as *U*_iso_(H) = 1.2*U*_eq_(carrier C atom).

### Crystal data

**Table:**

C_39_H_28_N_2_O	*Z* = 2
*M**_r_* = 540.63	*F*(000) = 568
Triclinic, *P*1	*D*_x_ = 1.245 Mg m^−^^3^
Hall symbol: -P 1	Mo *K*α radiation, λ = 0.71073 Å
*a* = 11.1723 (10) Å	Cell parameters from 82 reflections
*b* = 11.3487 (13) Å	θ = 4.6–12.5°
*c* = 13.2331 (15) Å	µ = 0.08 mm^−^^1^
α = 103.121 (9)°	*T* = 296 K
β = 105.170 (8)°	Prism, yellow
γ = 108.746 (8)°	0.55 × 0.28 × 0.24 mm
*V* = 1441.9 (3) Å^3^	

### Data collection

**Table:**

Bruker P4 diffractometer	*R*_int_ = 0.018
Radiation source: fine-focus sealed tube	θ_max_ = 26.4°, θ_min_ = 2.0°
graphite	*h* = −13→2
2θ/ω scans	*k* = −13→13
6892 measured reflections	*l* = −16→16
5865 independent reflections	3 standard reflections every 97 reflections
4471 reflections with *I* > 2σ(*I*)	intensity decay: 1%

### Refinement

**Table:**

Refinement on *F*^2^	Secondary atom site location: difference Fourier map
Least-squares matrix: full	Hydrogen site location: inferred from neighbouring sites
*R*[*F*^2^ > 2σ(*F*^2^)] = 0.042	H-atom parameters constrained
*wR*(*F*^2^) = 0.113	*w* = 1/[σ^2^(*F*_o_^2^) + (0.0461*P*)^2^ + 0.275*P*] where *P* = (*F*_o_^2^ + 2*F*_c_^2^)/3
*S* = 1.03	(Δ/σ)_max_ < 0.001
5865 reflections	Δρ_max_ = 0.19 e Å^−^^3^
380 parameters	Δρ_min_ = −0.14 e Å^−^^3^
0 restraints	Extinction correction: *SHELXL97* (Sheldrick, 2008), Fc^*^=kFc[1+0.001xFc^2^λ^3^/sin(2θ)]^-1/4^
0 constraints	Extinction coefficient: 0.0198 (18)
Primary atom site location: structure-invariant direct methods	

### Fractional atomic coordinates and isotropic or equivalent isotropic displacement parameters (Å^2^)

**Table:**

	*x*	*y*	*z*	*U*_iso_*/*U*_eq_	
C1	0.81457 (15)	0.23158 (15)	0.66331 (12)	0.0425 (3)	
O1	0.92972 (12)	0.26828 (14)	0.66240 (11)	0.0658 (4)	
C2	0.70743 (14)	0.24601 (14)	0.57791 (12)	0.0383 (3)	
C3	0.72166 (15)	0.24315 (15)	0.47594 (12)	0.0425 (3)	
H3A	0.7902	0.2224	0.4599	0.051*	
C4	0.63532 (15)	0.27069 (15)	0.39873 (12)	0.0407 (3)	
H4A	0.6459	0.2673	0.3309	0.049*	
C5	0.53212 (14)	0.30355 (13)	0.42020 (11)	0.0373 (3)	
C6	0.51690 (15)	0.30587 (15)	0.52197 (12)	0.0424 (3)	
H6A	0.4486	0.3270	0.5381	0.051*	
C7	0.60322 (15)	0.27683 (15)	0.59917 (12)	0.0416 (3)	
H7A	0.5914	0.2779	0.6663	0.050*	
N8	0.46589 (12)	0.34758 (12)	0.33981 (10)	0.0419 (3)	
C9	0.34487 (14)	0.34189 (13)	0.31471 (11)	0.0374 (3)	
C10	0.30371 (14)	0.40362 (14)	0.23057 (12)	0.0395 (3)	
C11	0.39664 (17)	0.46820 (17)	0.18792 (13)	0.0505 (4)	
H11A	0.4834	0.4696	0.2098	0.061*	
C12	0.3613 (2)	0.5302 (2)	0.11344 (15)	0.0614 (5)	
H12A	0.4245	0.5732	0.0858	0.074*	
C13	0.23276 (19)	0.52870 (18)	0.07989 (14)	0.0585 (4)	
H13A	0.2094	0.5712	0.0304	0.070*	
C14	0.14043 (18)	0.46438 (19)	0.11986 (15)	0.0614 (5)	
H14A	0.0534	0.4622	0.0968	0.074*	
C15	0.17516 (16)	0.40203 (17)	0.19474 (14)	0.0536 (4)	
H15A	0.1109	0.3585	0.2212	0.064*	
C16	0.24182 (14)	0.27839 (14)	0.36114 (11)	0.0372 (3)	
C17	0.20701 (16)	0.35573 (16)	0.43619 (13)	0.0473 (4)	
H17A	0.2458	0.4474	0.4564	0.057*	
C18	0.11442 (17)	0.29636 (18)	0.48108 (13)	0.0529 (4)	
H18A	0.0925	0.3485	0.5321	0.064*	
C19	0.05504 (17)	0.16072 (18)	0.45026 (14)	0.0547 (4)	
H19A	−0.0065	0.1214	0.4808	0.066*	
C20	0.08672 (18)	0.08337 (17)	0.37436 (16)	0.0575 (4)	
H20A	0.0454	−0.0083	0.3527	0.069*	
C21	0.18011 (16)	0.14185 (15)	0.33012 (14)	0.0477 (4)	
H21A	0.2015	0.0890	0.2792	0.057*	
C22	0.78337 (15)	0.17280 (14)	0.74811 (12)	0.0404 (3)	
C23	0.89013 (16)	0.20074 (16)	0.84510 (13)	0.0475 (4)	
H23A	0.9765	0.2609	0.8578	0.057*	
C24	0.87042 (17)	0.14101 (16)	0.92269 (13)	0.0513 (4)	
H24A	0.9428	0.1621	0.9875	0.062*	
C25	0.74229 (16)	0.04910 (14)	0.90414 (12)	0.0436 (3)	
C26	0.63567 (16)	0.02012 (16)	0.80788 (13)	0.0465 (4)	
H26A	0.5500	−0.0420	0.7944	0.056*	
C27	0.65529 (16)	0.08267 (15)	0.73147 (12)	0.0448 (3)	
H27A	0.5820	0.0642	0.6683	0.054*	
N28	0.72437 (15)	−0.02708 (13)	0.97419 (11)	0.0495 (3)	
C29	0.73136 (14)	0.02098 (14)	1.07383 (12)	0.0391 (3)	
C30	0.72464 (14)	−0.06736 (14)	1.14219 (12)	0.0409 (3)	
C31	0.7430 (2)	−0.18335 (17)	1.10693 (15)	0.0584 (4)	
H31A	0.7576	−0.2060	1.0405	0.070*	
C32	0.7397 (2)	−0.2648 (2)	1.17030 (18)	0.0717 (6)	
H32A	0.7527	−0.3419	1.1465	0.086*	
C33	0.7174 (2)	−0.23299 (19)	1.26838 (16)	0.0657 (5)	
H33A	0.7156	−0.2883	1.3107	0.079*	
C34	0.69800 (19)	−0.11972 (19)	1.30364 (15)	0.0598 (5)	
H34A	0.6823	−0.0985	1.3697	0.072*	
C35	0.70164 (16)	−0.03645 (16)	1.24098 (13)	0.0487 (4)	
H35A	0.6886	0.0404	1.2654	0.058*	
C36	0.74461 (14)	0.15897 (14)	1.12429 (12)	0.0386 (3)	
C37	0.85587 (15)	0.24739 (15)	1.21870 (13)	0.0452 (3)	
H37A	0.9195	0.2189	1.2534	0.054*	
C38	0.8732 (2)	0.37737 (17)	1.26174 (15)	0.0577 (4)	
H38A	0.9499	0.4367	1.3232	0.069*	
C39	0.7766 (2)	0.41837 (19)	1.21333 (17)	0.0657 (5)	
H39A	0.7879	0.5056	1.2423	0.079*	
C40	0.6634 (2)	0.3310 (2)	1.12217 (16)	0.0655 (5)	
H40A	0.5973	0.3588	1.0911	0.079*	
C41	0.64726 (18)	0.20148 (18)	1.07617 (14)	0.0513 (4)	
H41A	0.5716	0.1434	1.0134	0.062*	

### Atomic displacement parameters (Å^2^)

**Table:**

	*U*^11^	*U*^22^	*U*^33^	*U*^12^	*U*^13^	*U*^23^
C1	0.0422 (8)	0.0497 (9)	0.0425 (8)	0.0226 (7)	0.0182 (6)	0.0192 (7)
O1	0.0458 (7)	0.1042 (10)	0.0667 (8)	0.0347 (7)	0.0264 (6)	0.0504 (8)
C2	0.0402 (7)	0.0395 (7)	0.0392 (7)	0.0174 (6)	0.0164 (6)	0.0164 (6)
C3	0.0430 (8)	0.0527 (9)	0.0414 (8)	0.0253 (7)	0.0209 (7)	0.0179 (7)
C4	0.0414 (8)	0.0510 (8)	0.0347 (7)	0.0192 (7)	0.0181 (6)	0.0177 (6)
C5	0.0350 (7)	0.0376 (7)	0.0397 (7)	0.0130 (6)	0.0138 (6)	0.0161 (6)
C6	0.0424 (8)	0.0517 (8)	0.0437 (8)	0.0243 (7)	0.0220 (7)	0.0200 (7)
C7	0.0468 (8)	0.0499 (8)	0.0364 (7)	0.0229 (7)	0.0201 (6)	0.0186 (6)
N8	0.0394 (7)	0.0496 (7)	0.0434 (7)	0.0193 (6)	0.0172 (5)	0.0232 (6)
C9	0.0381 (7)	0.0367 (7)	0.0374 (7)	0.0149 (6)	0.0144 (6)	0.0123 (6)
C10	0.0392 (8)	0.0399 (8)	0.0383 (7)	0.0154 (6)	0.0130 (6)	0.0137 (6)
C11	0.0499 (9)	0.0678 (11)	0.0520 (9)	0.0316 (8)	0.0268 (8)	0.0320 (8)
C12	0.0657 (11)	0.0802 (13)	0.0621 (11)	0.0352 (10)	0.0355 (9)	0.0438 (10)
C13	0.0653 (11)	0.0697 (11)	0.0510 (9)	0.0329 (9)	0.0185 (8)	0.0343 (9)
C14	0.0475 (9)	0.0747 (12)	0.0651 (11)	0.0275 (9)	0.0112 (8)	0.0360 (10)
C15	0.0381 (8)	0.0637 (10)	0.0615 (10)	0.0174 (8)	0.0157 (7)	0.0329 (9)
C16	0.0337 (7)	0.0412 (7)	0.0382 (7)	0.0161 (6)	0.0121 (6)	0.0160 (6)
C17	0.0423 (8)	0.0450 (8)	0.0481 (8)	0.0151 (7)	0.0154 (7)	0.0089 (7)
C18	0.0487 (9)	0.0704 (11)	0.0422 (8)	0.0269 (8)	0.0210 (7)	0.0142 (8)
C19	0.0466 (9)	0.0738 (12)	0.0572 (10)	0.0240 (8)	0.0268 (8)	0.0376 (9)
C20	0.0563 (10)	0.0488 (9)	0.0772 (12)	0.0199 (8)	0.0311 (9)	0.0328 (9)
C21	0.0514 (9)	0.0426 (8)	0.0591 (9)	0.0223 (7)	0.0282 (8)	0.0205 (7)
C22	0.0453 (8)	0.0446 (8)	0.0380 (7)	0.0234 (7)	0.0167 (6)	0.0168 (6)
C23	0.0447 (8)	0.0502 (9)	0.0439 (8)	0.0159 (7)	0.0120 (7)	0.0189 (7)
C24	0.0524 (9)	0.0550 (9)	0.0376 (8)	0.0181 (8)	0.0059 (7)	0.0176 (7)
C25	0.0571 (9)	0.0407 (8)	0.0372 (7)	0.0219 (7)	0.0195 (7)	0.0147 (6)
C26	0.0461 (8)	0.0487 (9)	0.0441 (8)	0.0167 (7)	0.0170 (7)	0.0176 (7)
C27	0.0437 (8)	0.0519 (9)	0.0389 (7)	0.0208 (7)	0.0119 (6)	0.0173 (7)
N28	0.0639 (9)	0.0438 (7)	0.0414 (7)	0.0212 (6)	0.0180 (6)	0.0181 (6)
C29	0.0350 (7)	0.0417 (8)	0.0405 (8)	0.0147 (6)	0.0114 (6)	0.0180 (6)
C30	0.0374 (7)	0.0428 (8)	0.0423 (8)	0.0152 (6)	0.0111 (6)	0.0194 (6)
C31	0.0796 (12)	0.0550 (10)	0.0570 (10)	0.0361 (9)	0.0311 (9)	0.0284 (8)
C32	0.0982 (16)	0.0598 (11)	0.0783 (13)	0.0440 (11)	0.0347 (12)	0.0407 (10)
C33	0.0734 (12)	0.0606 (11)	0.0667 (12)	0.0231 (10)	0.0186 (10)	0.0418 (10)
C34	0.0614 (11)	0.0696 (12)	0.0512 (9)	0.0185 (9)	0.0243 (8)	0.0328 (9)
C35	0.0497 (9)	0.0516 (9)	0.0509 (9)	0.0203 (7)	0.0217 (7)	0.0246 (7)
C36	0.0397 (7)	0.0448 (8)	0.0427 (7)	0.0204 (6)	0.0213 (6)	0.0230 (6)
C37	0.0415 (8)	0.0470 (8)	0.0523 (9)	0.0195 (7)	0.0203 (7)	0.0201 (7)
C38	0.0665 (11)	0.0458 (9)	0.0624 (10)	0.0193 (8)	0.0317 (9)	0.0165 (8)
C39	0.1079 (16)	0.0549 (10)	0.0672 (12)	0.0482 (11)	0.0528 (12)	0.0327 (9)
C40	0.0996 (15)	0.0870 (14)	0.0656 (12)	0.0726 (13)	0.0506 (12)	0.0506 (11)
C41	0.0556 (10)	0.0696 (11)	0.0486 (9)	0.0373 (9)	0.0239 (8)	0.0322 (8)

### Geometric parameters (Å, °)

**Table:**

C1—O1	1.2225 (18)	C21—H21A	0.9300
C1—C22	1.490 (2)	C22—C27	1.391 (2)
C1—C2	1.496 (2)	C22—C23	1.393 (2)
C2—C7	1.395 (2)	C23—C24	1.379 (2)
C2—C3	1.394 (2)	C23—H23A	0.9300
C3—C4	1.377 (2)	C24—C25	1.392 (2)
C3—H3A	0.9300	C24—H24A	0.9300
C4—C5	1.396 (2)	C25—C26	1.383 (2)
C4—H4A	0.9300	C25—N28	1.4130 (19)
C5—C6	1.3964 (19)	C26—C27	1.384 (2)
C5—N8	1.4127 (18)	C26—H26A	0.9300
C6—C7	1.387 (2)	C27—H27A	0.9300
C6—H6A	0.9300	N28—C29	1.2784 (19)
C7—H7A	0.9300	C29—C30	1.4926 (19)
N8—C9	1.2813 (18)	C29—C36	1.498 (2)
C9—C10	1.4950 (19)	C30—C35	1.387 (2)
C9—C16	1.5033 (19)	C30—C31	1.392 (2)
C10—C15	1.382 (2)	C31—C32	1.380 (2)
C10—C11	1.393 (2)	C31—H31A	0.9300
C11—C12	1.383 (2)	C32—C33	1.375 (3)
C11—H11A	0.9300	C32—H32A	0.9300
C12—C13	1.381 (3)	C33—C34	1.370 (3)
C12—H12A	0.9300	C33—H33A	0.9300
C13—C14	1.361 (2)	C34—C35	1.390 (2)
C13—H13A	0.9300	C34—H34A	0.9300
C14—C15	1.388 (2)	C35—H35A	0.9300
C14—H14A	0.9300	C36—C37	1.389 (2)
C15—H15A	0.9300	C36—C41	1.391 (2)
C16—C21	1.386 (2)	C37—C38	1.384 (2)
C16—C17	1.391 (2)	C37—H37A	0.9300
C17—C18	1.390 (2)	C38—C39	1.373 (3)
C17—H17A	0.9300	C38—H38A	0.9300
C18—C19	1.376 (2)	C39—C40	1.375 (3)
C18—H18A	0.9300	C39—H39A	0.9300
C19—C20	1.375 (2)	C40—C41	1.389 (3)
C19—H19A	0.9300	C40—H40A	0.9300
C20—C21	1.387 (2)	C41—H41A	0.9300
C20—H20A	0.9300		
			
O1—C1—C22	119.75 (13)	C20—C21—H21A	119.7
O1—C1—C2	118.82 (13)	C27—C22—C23	118.10 (13)
C22—C1—C2	121.43 (13)	C27—C22—C1	123.37 (13)
C7—C2—C3	118.23 (13)	C23—C22—C1	118.30 (14)
C7—C2—C1	123.51 (13)	C24—C23—C22	121.35 (15)
C3—C2—C1	117.86 (13)	C24—C23—H23A	119.3
C4—C3—C2	120.68 (13)	C22—C23—H23A	119.3
C4—C3—H3A	119.7	C23—C24—C25	120.03 (14)
C2—C3—H3A	119.7	C23—C24—H24A	120.0
C3—C4—C5	121.35 (13)	C25—C24—H24A	120.0
C3—C4—H4A	119.3	C26—C25—C24	119.11 (14)
C5—C4—H4A	119.3	C26—C25—N28	119.71 (14)
C4—C5—C6	118.22 (13)	C24—C25—N28	120.61 (14)
C4—C5—N8	114.03 (12)	C25—C26—C27	120.64 (15)
C6—C5—N8	127.22 (13)	C25—C26—H26A	119.7
C7—C6—C5	120.31 (13)	C27—C26—H26A	119.7
C7—C6—H6A	119.8	C26—C27—C22	120.73 (14)
C5—C6—H6A	119.8	C26—C27—H27A	119.6
C6—C7—C2	121.20 (13)	C22—C27—H27A	119.6
C6—C7—H7A	119.4	C29—N28—C25	123.09 (13)
C2—C7—H7A	119.4	N28—C29—C30	117.26 (13)
C9—N8—C5	127.61 (12)	N28—C29—C36	123.77 (13)
N8—C9—C10	116.13 (13)	C30—C29—C36	118.97 (12)
N8—C9—C16	126.07 (13)	C35—C30—C31	118.81 (14)
C10—C9—C16	117.80 (12)	C35—C30—C29	121.70 (14)
C15—C10—C11	117.84 (14)	C31—C30—C29	119.49 (14)
C15—C10—C9	121.89 (13)	C32—C31—C30	120.19 (17)
C11—C10—C9	120.25 (13)	C32—C31—H31A	119.9
C12—C11—C10	120.72 (15)	C30—C31—H31A	119.9
C12—C11—H11A	119.6	C33—C32—C31	120.56 (18)
C10—C11—H11A	119.6	C33—C32—H32A	119.7
C13—C12—C11	120.39 (16)	C31—C32—H32A	119.7
C13—C12—H12A	119.8	C34—C33—C32	119.89 (16)
C11—C12—H12A	119.8	C34—C33—H33A	120.1
C14—C13—C12	119.44 (15)	C32—C33—H33A	120.1
C14—C13—H13A	120.3	C33—C34—C35	120.21 (17)
C12—C13—H13A	120.3	C33—C34—H34A	119.9
C13—C14—C15	120.52 (16)	C35—C34—H34A	119.9
C13—C14—H14A	119.7	C30—C35—C34	120.34 (16)
C15—C14—H14A	119.7	C30—C35—H35A	119.8
C10—C15—C14	121.07 (15)	C34—C35—H35A	119.8
C10—C15—H15A	119.5	C37—C36—C41	118.92 (14)
C14—C15—H15A	119.5	C37—C36—C29	120.45 (13)
C21—C16—C17	118.85 (14)	C41—C36—C29	120.62 (14)
C21—C16—C9	120.62 (13)	C38—C37—C36	120.79 (15)
C17—C16—C9	120.53 (13)	C38—C37—H37A	119.6
C16—C17—C18	120.17 (15)	C36—C37—H37A	119.6
C16—C17—H17A	119.9	C39—C38—C37	119.74 (18)
C18—C17—H17A	119.9	C39—C38—H38A	120.1
C19—C18—C17	120.25 (15)	C37—C38—H38A	120.1
C19—C18—H18A	119.9	C38—C39—C40	120.28 (17)
C17—C18—H18A	119.9	C38—C39—H39A	119.9
C20—C19—C18	119.98 (15)	C40—C39—H39A	119.9
C20—C19—H19A	120.0	C39—C40—C41	120.41 (17)
C18—C19—H19A	120.0	C39—C40—H40A	119.8
C19—C20—C21	120.12 (16)	C41—C40—H40A	119.8
C19—C20—H20A	119.9	C40—C41—C36	119.79 (17)
C21—C20—H20A	119.9	C40—C41—H41A	120.1
C16—C21—C20	120.60 (15)	C36—C41—H41A	120.1
C16—C21—H21A	119.7		
			
O1—C1—C2—C7	−144.52 (16)	O1—C1—C22—C27	−152.96 (16)
C22—C1—C2—C7	36.0 (2)	C2—C1—C22—C27	26.6 (2)
O1—C1—C2—C3	28.0 (2)	O1—C1—C22—C23	21.4 (2)
C22—C1—C2—C3	−151.51 (14)	C2—C1—C22—C23	−159.09 (14)
C7—C2—C3—C4	0.3 (2)	C27—C22—C23—C24	0.2 (2)
C1—C2—C3—C4	−172.64 (14)	C1—C22—C23—C24	−174.49 (14)
C2—C3—C4—C5	0.7 (2)	C22—C23—C24—C25	1.0 (3)
C3—C4—C5—C6	−1.0 (2)	C23—C24—C25—C26	−0.7 (2)
C3—C4—C5—N8	171.19 (13)	C23—C24—C25—N28	170.63 (14)
C4—C5—C6—C7	0.4 (2)	C24—C25—C26—C27	−0.8 (2)
N8—C5—C6—C7	−170.67 (14)	N28—C25—C26—C27	−172.23 (14)
C5—C6—C7—C2	0.6 (2)	C25—C26—C27—C22	2.0 (2)
C3—C2—C7—C6	−0.9 (2)	C23—C22—C27—C26	−1.7 (2)
C1—C2—C7—C6	171.58 (14)	C1—C22—C27—C26	172.67 (14)
C4—C5—N8—C9	154.35 (15)	C26—C25—N28—C29	−115.96 (18)
C6—C5—N8—C9	−34.3 (2)	C24—C25—N28—C29	72.8 (2)
C5—N8—C9—C10	176.47 (13)	C25—N28—C29—C30	−173.82 (14)
C5—N8—C9—C16	−4.1 (2)	C25—N28—C29—C36	6.7 (2)
N8—C9—C10—C15	178.78 (15)	N28—C29—C30—C35	−165.71 (15)
C16—C9—C10—C15	−0.7 (2)	C36—C29—C30—C35	13.8 (2)
N8—C9—C10—C11	−3.0 (2)	N28—C29—C30—C31	14.9 (2)
C16—C9—C10—C11	177.57 (14)	C36—C29—C30—C31	−165.62 (15)
C15—C10—C11—C12	1.0 (2)	C35—C30—C31—C32	−0.7 (3)
C9—C10—C11—C12	−177.38 (15)	C29—C30—C31—C32	178.75 (17)
C10—C11—C12—C13	−0.2 (3)	C30—C31—C32—C33	0.4 (3)
C11—C12—C13—C14	−0.7 (3)	C31—C32—C33—C34	0.2 (3)
C12—C13—C14—C15	0.7 (3)	C32—C33—C34—C35	−0.4 (3)
C11—C10—C15—C14	−0.9 (3)	C31—C30—C35—C34	0.4 (2)
C9—C10—C15—C14	177.43 (16)	C29—C30—C35—C34	−179.01 (15)
C13—C14—C15—C10	0.0 (3)	C33—C34—C35—C30	0.1 (3)
N8—C9—C16—C21	−72.1 (2)	N28—C29—C36—C37	−120.14 (17)
C10—C9—C16—C21	107.31 (16)	C30—C29—C36—C37	60.38 (18)
N8—C9—C16—C17	107.75 (18)	N28—C29—C36—C41	58.6 (2)
C10—C9—C16—C17	−72.83 (18)	C30—C29—C36—C41	−120.93 (15)
C21—C16—C17—C18	1.6 (2)	C41—C36—C37—C38	−2.8 (2)
C9—C16—C17—C18	−178.23 (14)	C29—C36—C37—C38	175.92 (14)
C16—C17—C18—C19	−1.0 (2)	C36—C37—C38—C39	2.6 (2)
C17—C18—C19—C20	−0.3 (3)	C37—C38—C39—C40	−0.3 (3)
C18—C19—C20—C21	1.0 (3)	C38—C39—C40—C41	−1.8 (3)
C17—C16—C21—C20	−0.9 (2)	C39—C40—C41—C36	1.6 (3)
C9—C16—C21—C20	178.93 (15)	C37—C36—C41—C40	0.7 (2)
C19—C20—C21—C16	−0.4 (3)	C29—C36—C41—C40	−178.01 (14)
